# An automated module for atomicabsorption analysis of reduced volatile elements

**DOI:** 10.1155/S1463924682000170

**Published:** 1982

**Authors:** Brian W. Renoe

**Affiliations:** Departments of Pathology and of Chemistry University of Virginia Charlottesville Virginia 22908 USA


Journal of Automatic Chemistry, Volume 4, Number 2 (April–June 1982), pages 61–64

### An automated module for atomic-absorption analysis of reduced volatile elements

**Brian W. Renoe**

*Departments of Pathology and of Chemistry, University of Virginia, Charlottesville, Virginia 22908, USA*

### Introduction

The atomic-absorption determination of elements that can be reduced to volatile forms through the use of reducing agents, such as sodium borohydride, has become an accepted means of analysis for arsenic and mercury, and application to other elements is possible [1–7]. In adapting this technique at the University of Virginia Clinical Chemistry Laboratory for the determination of arsenic and mercury in serum, urine and tissue, it was noted that the precision of the results was highly operator-dependent, ranging from 2% to 10% or more relative standard deviation. To eliminate this uncontrolled variance the options were either to develop an automated approach, such as that presented by Dennis and Porter [8] using continuous-flow principles, or to modify the manual approach to improve analytical integrity. As the anticipated number of samples was small (between one and six a week), and because of the complexity of the continuous-flow manifold for handling the gaseous hydride species, the second approach was chosen. To improve precision the various steps in the procedure were examined in detail so that their individual contribution to total imprecision could be determined. The analysis steps for the manual procedure are: switching the purge gas through the sample in the reaction vessel, adding the acid diluent to the sample, initiating the atomic-absorption measurement cycle, adding the basic sodium borohydride reductant solution, and resetting the atomic-absorption instrument and the purge-gas flow. With these steps identified, and the contribution of each one to the total imprecision estimated, it was decided that a logic timing control unit that could be used with a commercial atomic-absorption instrument should be constructed. This module would remove the variable timing and solution volume dependence of each step and provide the consistency necessary for better precision of the total analysis.

The module described here allows the operator to put a liquid sample manually into the reaction vessel, then initiate a fixed sequence of analysis steps, which culminates in an absorbance or a concentration reading for the element. The module has been used with an Instrumentation Laboratory Model 751 atomic-absorption instrument (Analytical Instrument Division, Instrumentation Laboratory Inc., Wilmington, Massachusetts 01720, USA). The module has significantly improved the efficiency of analysis for arsenic and mercury compared to the manually manipulated and timed procedure. The system is easily adaptable to the determination of other metals.

### Apparatus

The module, which is best described as a timer/sequencer, is shown in diagram form in figure 1. The module is constructed of transistor-transistor logic (TTL) integrated circuits, which provide the step sequencing required. It is electronically interfaced to the reagents, purge gas and the atomic-absorption instrument through optically isolated components. The relative orientation of the plumbing necessary for the automated module to control the analysis steps is shown in figure 2. The module controls the analysis steps in such a manner that the operator has only to manually put the liquid sample into the interchangeable reaction vessel and then push the start button. This initiates a fixed sequence of analysis steps, finishing with the presentation of the element's absorbance or a concentration reading.

As shown in figure 2, the plumbing manifold has tubing connections to allow the purge gas (compressed air or argon) to be switched either directly to the fused silica flow-tube, or through the reaction vessel and then to the fused silica flow-tube. The remainder of the plumbing is used for connecting acid and sodium borohydride reagents to the reaction vessel. The reaction solution is continuously mixed by the purge-gas stream during analysis.

Nichrome wire is used to inductively heat the flow tube, alternatively the tube can be placed in an air/acetylene flame. The uniformity of heating provided by the flame and its ease of use makes this method preferable.

Compressed air and argon are used as purge gases. It is necessary to have oxidizing conditions in the flow tube to convert the elemental hydrides to the free elements. Generally, with uniform heating and a small amount of oxidizing gas, this oxidization step can be controlled to yield a uniform conversion.

Details of the electronic circuitry are shown in figure 3. For simplicity's sake the power-supply and ground connections are not shown, except where they are necessary to specify a logic level in the TTL components, or to complete the circuitry for the discrete components used. There are two power-supplies in the module a +5 V DC supply for the TTL components, and a +12 V DC supply for the solenoid valves. Both supplies are rectified and filtered with three-pin regulators (see Components list in table 1).

The sequence of control events is initiated by closing the contacts on the start push-button (Start P.B. in figures 1 and 3). This clears the data latch (output Q = O) (one half of a 7474 D-type positive-edge triggered flip-flop), which in turn enables the binary counter (7493) and the 1-of-16 data distributor (74154 4-line-to-16-line decoder/demultiplexer). This also sets a data latch (output Q = 1) (the other half of the 7474) to initiate the argon flow through the reaction vessel. The argon is switched by the three-way solenoid valve which is actuated through the optical isolator/pass transistor circuit detailed in figure 3.

After initiation the clock pulses from the timer-astable (555 timer) are counted by the binary counter which gives 16 distinct output states, thus allowing timing of different events. The time duration of each event is controlled by the frequency of the astable, with both 1 and 4 s having been used for the hydride-generator procedures. One second yields a 16 s total cycle time (used for mercury); 4 s allows for approximately 1 min. total

0142 0453/82/0402 0061 $05.00 © 1982 Taylor & Francis Ltd

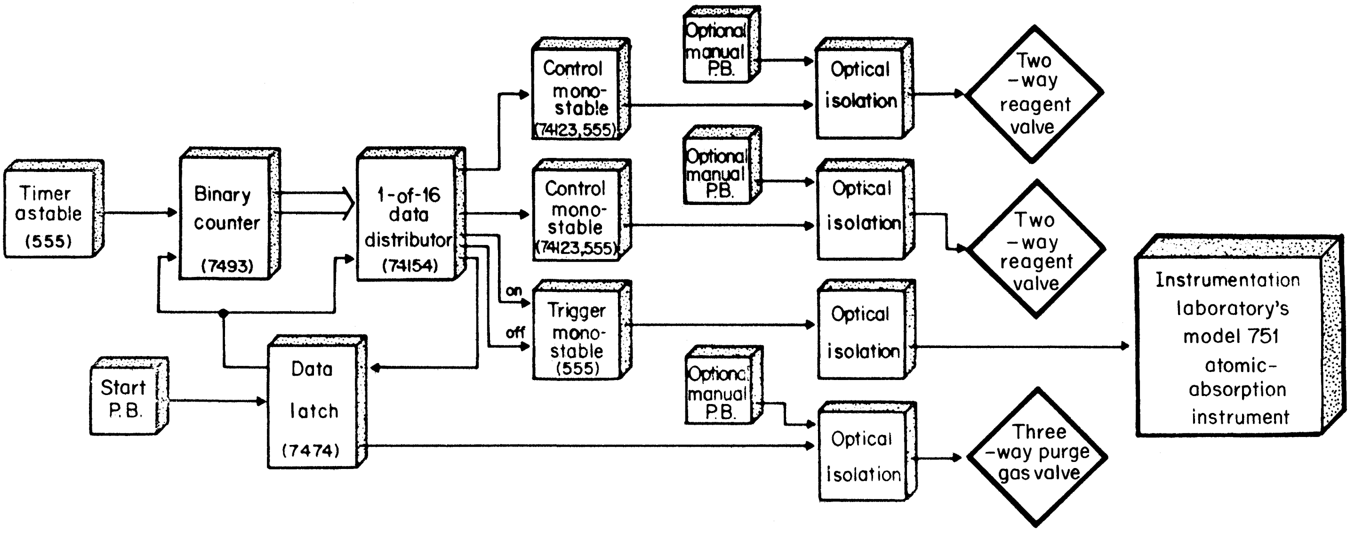

*Figure 1. Block diagram of electronics for hydride generation atomic-absorption analysis.*

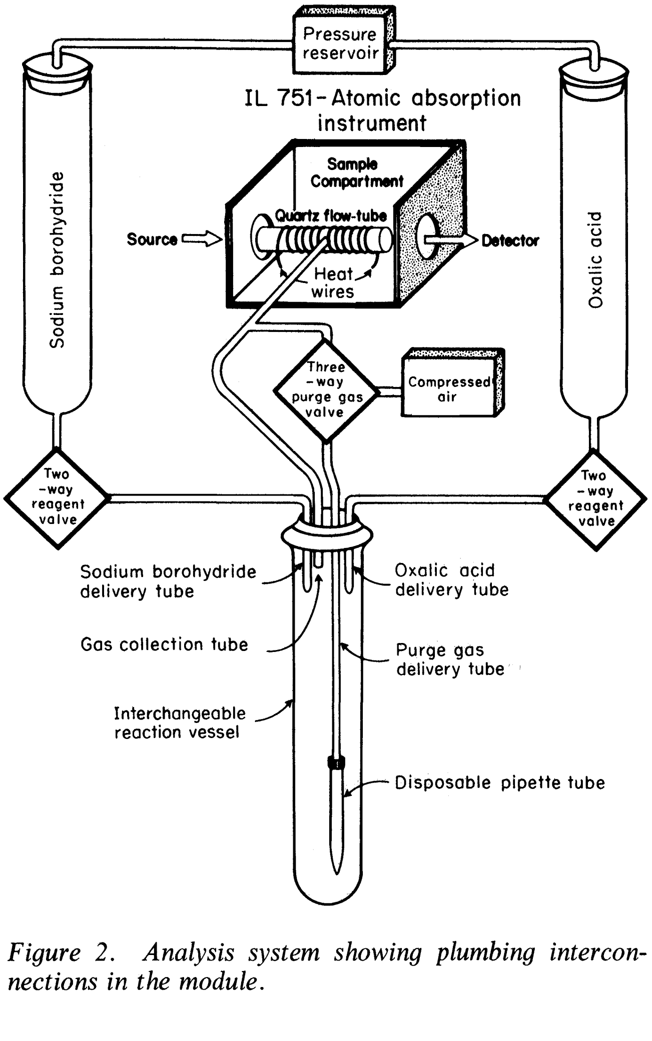

*Figure 2. Analysis system showing plumbing interconnections in the module.*

cycle time (used for arsenic, selenium etc.).

The binary counter is connected to the four inputs of the data distributor. The data distributor outputs provide the event-sequencing function. The outputs are '$\overline{0}$' to '$\overline{15}$' and selection of appropriate outputs will allow the desired event sequencing. As shown in figure 3, the atomic-absorption instrument-read cycle (the second analytical event after switching the purge gas through the reaction vessel) begins as the '$\overline{2}$' output of the data distributor goes low. This low signal causes a closing of the solid-state relay, which is in parallel with the panel 'READ' button on the IL 751, and will remain in the data-acquisition state until it is turned off (a second closure of the relay) when the '$\overline{14}$' output of the data distributor goes low.

The other two events which must be correctly and reproducibly sequenced for the hydride analysis procedure are the addition of the acid diluent and of the sodium borohydride reductant solution. Addition of the acid diluent is initiated when output '$\overline{4}$' of the data distributor goes low, triggering the first monostable (one half of a 74123 dual retriggerable monostable multivibrator) which presents a 1 ms wide pulse to the second monostable (555 timer) to trigger its output. The pulse width of the 555 timer monostable, which is controlled by the 10 $\mu$F capacitor and the 500 k$\Omega$ potentiometer, can be adjusted from approximately 1 s to 10 s. The pulse opens the reagent control valve for the oxalic acid and holds it open for the time interval specified by the pulse width. The sodium borohydride reductant solution is added as the fourth event of the analysis cycle—when the '$\overline{6}$' output of the data distributor goes low. The volume of this solution is operator controlled in the same way as the acid diluent volume.

The final event controlled by the timer/sequencer module is the resetting of the system to await another start command from the operator. This is accomplished by resetting the 7474 data latches to disable the counter and data distributor and to switch the purge gas directly through the flow tube, bypassing the reaction vessel.

### Analytical results

The module described has been used to determine arsenic and mercury in urine samples submitted to the University of Virginia Clinical Chemistry Laboratory. The analysis protocol is to transfer 1 ml of each urine sample into the interchangeable reaction vessels, add one drop of antifoam reagent (Antifoam B, Beckman Instruments Inc., Brea, California 92621, USA) and fit the reaction vessel to the module. The operator then starts the process described above, which requires about 1 min. After the results are recorded from the atomic-absorption instrument, the operator fits a clean disposable pipette tip to the purge-gas outlet, a fresh reaction vessel, and initiates the next analysis. In this way, the total analysis time for three standards and six patient samples is less than 15 min.

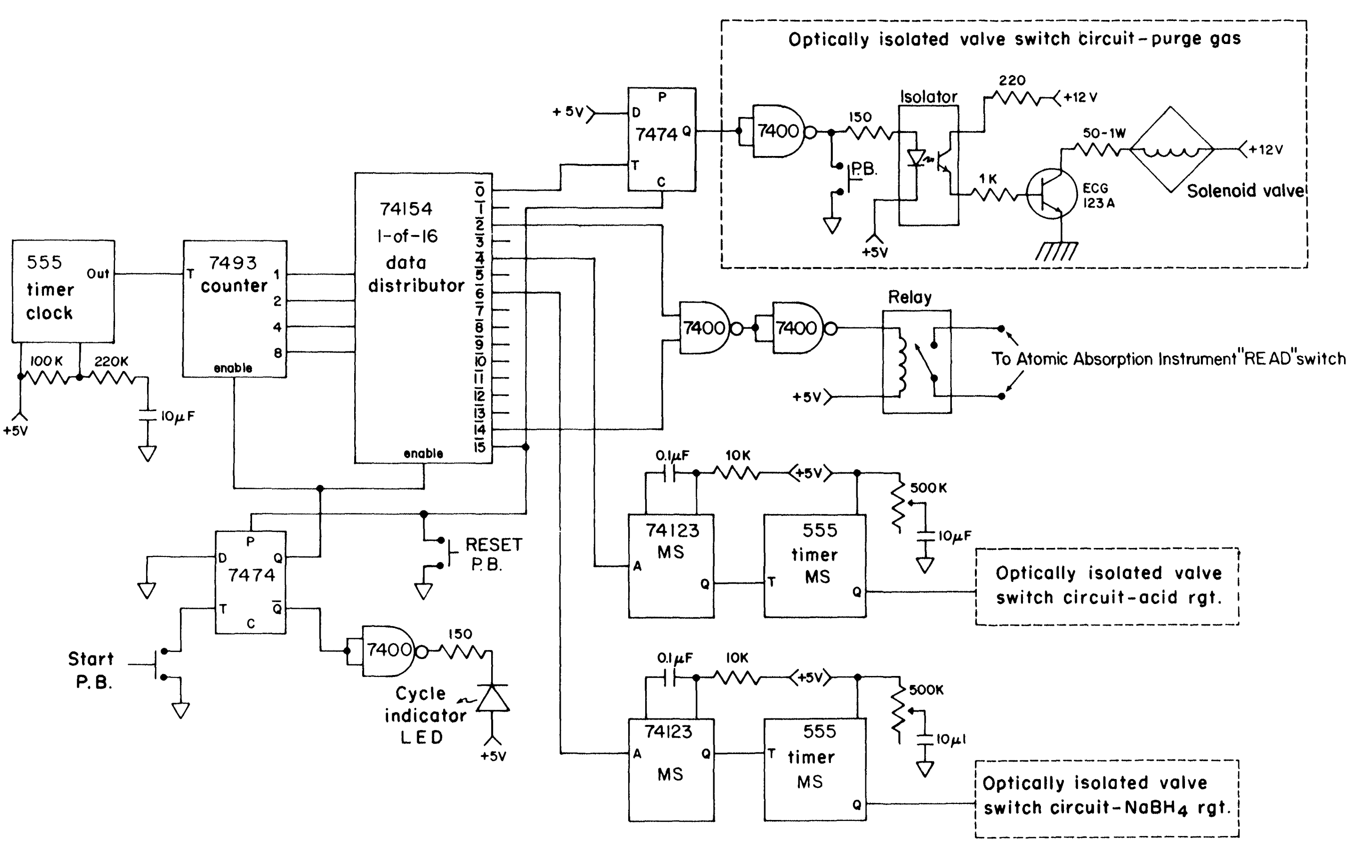

*Figure 3. Detailed circuit diagram of the timing sequencer.*

*Table 1. Module components list.*

| *Description* | *Number* | *Quantity* |
|---|---|---|
| Transistor-transistor logic (TTL) circuits | | |
| Quadrupole two-input positive nand gates | 7400 | 2 |
| Dual D-type positive-edge triggered flip-flop | 7474 | 1 |
| Binary four-bit counter | 7493 | 1 |
| Dual retriggerable monostable multivibrators with clear | 74123 | 1 |
| 4-line-to-16-line decoders/ demultiplexers | 74154 | 1 |
| Timer, astable or monostable | 555 | 3 |
| Photon-coupled isolator (General Electric, Photo-Darlington Amplifier) | H11B1 | 3 |
| Solid-state relay (Magnacraft) | W117 DIP-9 | 1 |
| Power transistor | ECG 123A | 3 |
| Potentiometers | 500 Kohm | 2 |
| Capacitors | (see figure 3) | |
| Resistors | (see figure 3) | |
| Light-emitting diodes (LEDs) | (See figure 3) | |
| Push buttons | Normally open | 5 |
| Power-supplies | | |
| 12 V DC (transformer, rectifier bridge, 2–0·33 μF filter capacitor and 7812 three-pin regulator) | | 1 |
| 5 V DC (transformer, recifier bridge, 0·33 μF filter capacitor, and 7805 three-pin regulator) | | 1 |

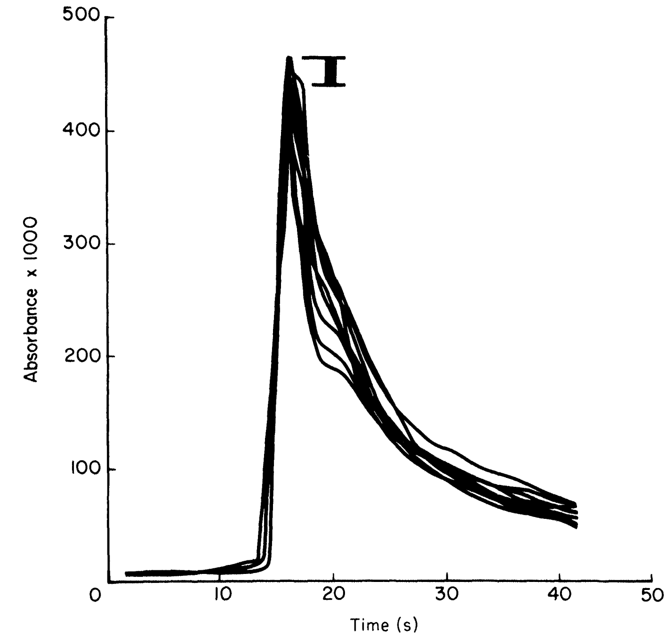

*Figure 4. Atomic-absorption time response using the timing sequencer, run 10 times for urine samples with about 0·2 μg/ml of added arsenic.*

The precision of the results thus obtained is illustrated for arsenic in figure 4, which shows 10 analysis cycles of a urine samples containing approximately 0·2 μg/ml of added arsenic. The total range of the peak absorbance from the 10 runs is indicated by the error bars near the top of the peak. The relative standard deviation for the 10 runs was less than 2%, using the peak absorbance. Mercury results were equally precise.

Inter-run precision has been documented over 30 separate days to be 3%. The accuracy of the apparatus/method was determined using a recovery approach with both sodium arsenate and sodium arsenite added to urine to yield a final concentration of 0·05 μg/ml arsenic in two separate experiments. The recoveries of added arsenic were 98% and 99% respectively.

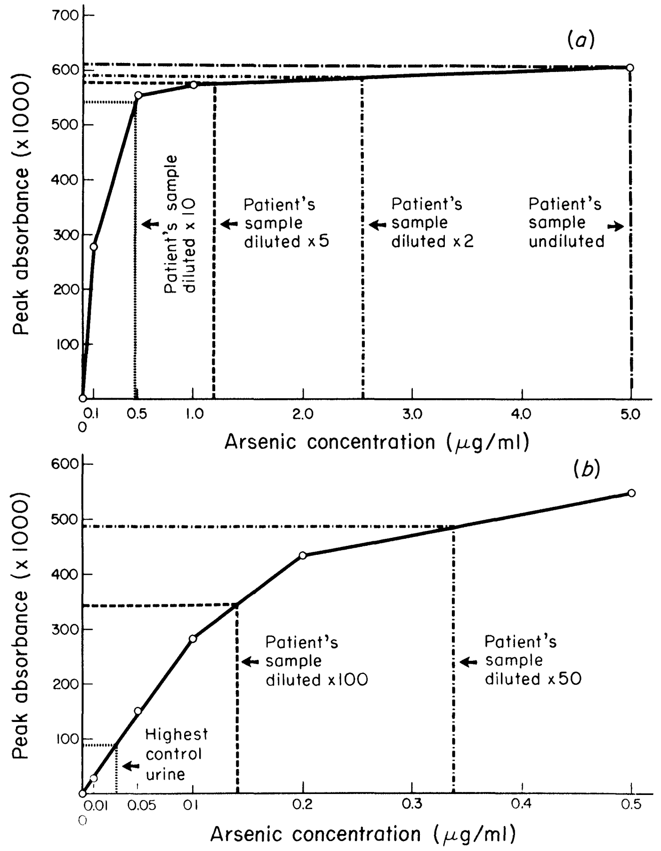

*Figure 5. System response to high concentration of arsenic, from the urine of a patient poisoned by arsenic.*

Figure 5 demonstrates the dynamic range of the instrument system, again for arsenic in urine. This figure reveals the peak absorbance measured from the arsenic in the urine of a patient who ingested a self-administered fatal quantity of sodium arsenate/arsenite (rat poison). The initial 1 ml aliquot of urine yielded a peak absorbance of 0·610 (see figure 5 [*a*]) which is beyond the linear range of the atomic-absorption measurement (approximately 0 to 0·1 μg/ml). The patient's urine was subsequently diluted two, five, 10, 50 and 100 times and reassayed using the semi-automatic system. Figure 5 shows the decrease in the peak absorbances as expected for the atomic-absorbance measurements, with the 100 times dilution approaching the linear response region of the atomic absorption measurement (see figure 5 [*b*]). The figure shows a high negative (non-toxic) control urine run with patient specimens for comparison (the reference range is 0–0·5 μg/ml arsenic).

The analytical precision possible with this module, and the ease of operation without manual manipulations, should allow it to be used in many laboratories where elements are determined by atomic absorption coupled to hydride generation of reduced volatile metal species.

### References

1. Heintges, M. G., Toffaletti, J., and Savory, J., *Clinical Chemistry*, **23** (1977), 1161.
2. Sharma, D. C. and Davis, P. S., *Clinical Chemistry*, **25** (1979), 769.
3. Fiorino, J. A. and Capar, S. G., *Analytical Chemistry*, **48** (1976), 120.
4. Brodie, K. G., *American Laboratory* (June 1979), 58.
5. Fernandez, F. J., *Atomic Absorption Newsletter*, **12** (1973), 93.
6. Thompson, K. C. and Thompson, D. R., *Analyst*, **99** (1974), 595.
7. Vijan, P. N., *American Laboratory* (August 1979), 32.
8. Dennis, A. L. and Porter, D. G., *Journal of Automatic Chemistry*, **2** (1980), 134.

### Conference announcement

*Flow Analysis II–an International Symposium*

Flow Analysis II will be held at the Hotel Sparta in Lund from 18–21 June. The organizers are the Analytical Section of the Swedish Chemical Society and they intend the scope and aims of the conference to be the same as the first Flow Analysis symposium, which was held in Amsterdam in 1979. Their scientific programme has been designed to reflect the rapid development and increasing importance of continuous flow analysis in recent years. The programme includes invited plenary lectures and submitted research papers which will be presented as lectures or poster sessions. The principle topics to be considered are: the theory of flow analysis; instrument design for continuous segmented and unsegmented flow analysis and flow injection analysis; new detector systems; possibilities for complete automation; and applications in industrial, environmental and clinical analysis. The official language at the meeting will be English and the participation fee is 600 krona. The papers will be published in a special issue of *Analytica Chimica Acta*.

*Further information from 'Flow Analysis II', c/o The Swedish Chemical Society, Upplandsgatan 6A, 1 tr, S–111 23 Stockholm.*

